# Sexual Perversion—II

**Published:** 1909-01-30

**Authors:** 


### Mental Diseases.

### SEXUAL PERVERSION—II.

After eliminating any anatomical or pathological abnormality which might possibly be a cause of sexual perversion, efforts towards its prevention fall under the heading of education. It is impossible here to do more than hint briefly at the ends to be aimed at in childhood that the adult may be healthy in mind and body.

Three of the most important objects in the mental education of the child are, to sustain interest, to cultivate powers of attention, and to establish self-control. These three objects must be as carefully kept in mind during the hours of recreation as during those of lessons; games, and athletics, when properly organised, are of very great value in the mental as well as in the physical education. Though the tastes and disposition of the adult depend to a large extent on the moral, intellectual, and artistic atmosphere of the child's home, every child has a natural predisposition to be interested in some particular subjects more than others.

Any natural bent that the child may display in any of the sciences, arts, crafts, and so forth, should never be ignored, as they may not only give valuable indications of the future sphere of work, but from them also arise those useful safety valves of later life —the various hobbies. In childhood, too, the most important habits are formed, and it is generally from the oft-repeated teachings of this period of life that many of the most fixed beliefs of after years are derived. It is urgent, then, that the teachings of physiology should be closely followed in early education, in order that the first formed and most fundamental habits may be such as to conduce to the future normality of the individual.

At puberty the child should receive some instruction in the physiology of sex, and be warned of the dangers to mental and physical health which may arise from bad habits. Should the parents be disinclined to give this instruction, the task should be entrusted to some competent person of the same sex as the child, who will give it fully with tact and discretion.

Were this advice always followed, the knowledge acquired would enable the child to avoid habits which may be contracted owing to ignorance, and would tend to put a stop to much speculative thought and conversation on this subject in youth which the lack of correct information gives rise to. Many educational authorities advocate early instruction in natural history, beginning with botany and passing on to zoology, including the physiology of propagation, so that by the time the child reaches the age of puberty this instruction will have given the requisite accurate knowledge.

Cases of sexual perversion which come under the physician for treatment can be divided into two classes (1) those who will actively co-operate in the treatment and (2) those who will not do so. In dealing with those of the first class, any physical cause which may be found should, of course, be removed if possible. Drugs may at times be indicated, or help to re-establish the general health. Diet and mode of life will also require attention. The main part of the treatment, however, will be rather psychological, directed towards helping the patient to increased inhibition; and this should be undertaken by someone with some influence over the patient, and, above all, with much tact and knowledge of the world.

In dealing with young people the campaign is usually opened with some "straight talks," in which it is attempted to bring home to them the evil consequences of their conduct. The line of argument used in these "talks" should be based on reason and scientific teaching, to show the effects of bad sexual habits on the mental and physical health and on the social life of the individual. The "damnation here and hereafter" argument should be used with the greatest caution to the young and impressionable, as it may end by helping to cause trouble worse than the habit. The harm accruing to some who throw off their incipient weakness from this pernicious teaching may be seen in many cases of melancholia with delusions of unworthiness which come into our asylums.

The removal of the patient from any bad companions, whose influence is contaminating, is essential, and may be advisable from the surroundings in which the habits have started. Foreign travel can at times be recommended, provided there is someone to be a companion to prevent the patient from drifting. Still better is hard work in the open air, with hard living. The advisability of pressing on marriage requires mature consideration, as serious disaster may follow the adoption of this course. Hypnotism has been practised in these cases, but authorities seem to doubt its value, though suggestion is of use. If the perversion seems to be a symptom of acute mental disorder, early certification is the safest course for the patient, friends, and doctor.

To treat cases that fall in the second class is always difficult, and often impossible, especially if all attempts to induce the patient to desire reform are in vain, as it is only by their own endeavours that they can be cured. In conclusion, those cases of this class whose perversion inclines them to acts of a criminal character require a word. One has the alternatives of regarding them either as criminal or as insane—criminal in that their acts render them liable to punishment under our penal code, or insane in that their feelings and desires are abnormal, and that their self-restraint is too deficient to allow them to regulate their conduct to the ordinary standard of convention, in this case prescribed by law. Which of these views should be held is a matter of opinion, and to a large extent must depend on the particular case. It also involves a discussion on the dividing line between sanity and insanity. The question cannot be discussed here, but from a practical point of view the desirability of treating such cases under certificates rather than in prison seems to have more in its favour than the mere avoidance of scandal and of the publication of unsavoury details.

